# Renalase Prevents Renal Fibrosis by Inhibiting Endoplasmic Reticulum Stress and Down-Regulating GSK-3β/Snail Signaling

**DOI:** 10.7150/ijms.82192

**Published:** 2023-03-21

**Authors:** Yiru Wu, Yu Bai, Yiduo Feng, Qidong Zhang, Zongli Diao, Wenhu Liu

**Affiliations:** 1 Department of Nephrology, Beijing Friendship Hospital, Capital Medical University, No. 95 Yong An Road, Xi Cheng District, Beijing 100050, P. R. China.; 2 Department of Nephrology, Beijing Tiantan Hospital, Capital Medical University, No.119 South Fourth Ring Road West, Fengtai District, Beijing,100070, P. R. China.

**Keywords:** Renal fibrosis, Renalase, GSK-3β/Snail, Endoplasmic reticulum stress, Chronic kidney disease.

## Abstract

**Background:** Treating renal fibrosis is crucial to delaying chronic kidney disease. The glycogen synthase kinase-3β (GSK-3β)/Snail pathway regulates renal fibrosis and Renalase can ameliorate renal interstitial fibrosis. However, it is not clear whether GSK-3β/Snail signaling affects Renalase action. Here, we explored the role and mechanism of GSK-3β/Snail in the anti-fibrosis action of Renalase.

**Materials and methods:** We used mice with complete unilateral ureteral obstruction (UUO) and human proximal renal tubular epithelial (HK-2) cells with transforming growth factor-β1 (TGF-β1)-induced fibrosis to explore the role and regulatory mechanism of the GSK-3β/Snail pathway in the amelioration of renal fibrosis by Renalase.

**Results:** In UUO mice and TGF-β1-induced fibrotic HK-2 cells, the expression of p-GSK-3β-Tyr216/p-GSK-3β-Ser9, GSK-3β and Snail was significantly increased, and endoplasmic reticulum (ER) stress was activated. After Renalase supplementation, fibrosis was alleviated, ER stress was inhibited and p-GSK-3β-Tyr216/p-GSK-3β-Ser9, GSK-3β and Snail were significantly down-regulated. The amelioration of renal fibrosis by Renalase and its inhibitory effect on GSK-3β/Snail were reversed by an ER stress agonist. Furthermore, when an adeno-associated virus or plasmid was used to overexpress GSK-3β, the effect of Renalase on delaying renal fibrosis was counteracted, although ER stress markers did not change.

**Conclusion:** Renalase prevents renal fibrosis by down-regulating GSK-3β/Snail signaling through inhibition of ER stress. Exogenous Renalase may be an effective method of slowing or stopping chronic kidney disease progression.

## Introduction

With aging populations and increasing rates of diabetes and hypertension, the prevalence of chronic kidney diseases (CKD) is constantly rising and now occurs in 10%-13% of the adult population worldwidely 1, 2. When CKD progress to end stage renal disease (ESRD), patient survival depends on renal replacement therapy^3^, which is costly and a great burden to patients' families and society. Renal fibrosis, especially renal tubulointerstitial fibrosis, is a sign and common feature of various progressive CKD. Renal fibrosis is also the main determinant and a reliable prognostic indicator of renal insufficiency and mainly manifests as sclerosis, renal tubular atrophy and renal interstitial fibrosis^4^. Renal fibrosis is considered to result from the failure of various kidney injuries to heal^5^. Delaying renal fibrosis is essential for delaying CKD progression; however, no effective drugs against renal fibrosis are currently available^6^.

Glycogen synthase kinase-3β (GSK-3β), a serine/threonine (Ser/Thr) protein kinase, is involved in a variety of processes, including extracellular matrix accumulation and epithelial-mesenchymal transformation (EMT) and, therefore, plays a role in fibrosis in various organs/tissues, including the heart, lungs, liver and kidney^7^. At present, the relationship between GSK-3β and fibrosis is not fully understood. Although many studies have shown that GSK-3β can alleviate organ fibrosis, other studies have shown that GSK-3β can promote fibrosis^7^. GSK-3β can mediate phosphorylation of substrates, including Snail, a zinc finger transcription factor^7^ and the GSK-3β/Snail pathway can regulate Renal fibrosis^8^, ^9^. We previously confirmed that Renalase can alleviate renal interstitial fibrosis by inhibiting the ERK pathway and oxidative stress^10^, ^11^. However, it is not clear whether GSK-3β/Snail is involved in this process. In this study, we explored the role of GSK-3β/Snail in the anti-fibrosis action of Renalase and investigated the underlying molecular mechanism.

## Materials and Methods

### Animal Experiments

#### Animal Feeding

Five-week-old male C57BL/6 mice (weighing approximately 16-18g) were obtained from The Institute of Experimental Animal Research, Chinese Academy of Medical Sciences. Prior to surgery, mice were housed with a 12-hour light-dark cycle and given free access to standard mouse food and water for two weeks.

#### Animal Grouping and Treatment

Animals were randomly assigned to the following groups (n = 5 per group): (1) Sham+Ad-β-gal; (2) Sham+Ad-Renalase; (3) Unilateral ureteral obstruction (UUO)+Ad-β-gal; (4) UUO+Ad-Renalase; (5) UUO+Ad-Renalase+tunicamycin (TM); (6) UUO+Ad-Renalase+4-phenylbutyric acid (4-PBA); (7) UUO+Ad-Renalase+AAV-GSK-3β; (8) UUO+Ad-Renalase+Ad-GSK-3β-RNAi. The mice was treated as follows: Ad-β-gal: lateral renal vein injected with 1.0×10^10^ plaque forming units (PFU) of control adenovirus during surgery; Ad-Renalase: lateral renal vein injected with 1.0×10^10^ PFU adenovirus-Renalase during surgery;TM: intraperitoneally injected with 1 mg/kg TM during surgery; 4-PBA: intraperitoneally injected with 300 mg/kg 4-PBA during surgery; AAV-GSK-3β: tail vein injected with 1.0×10^12^ PFU adeno-associated virus-GSK-3β one week before surgery; Ad-GSK-3β-RNAi: lateral renal vein injected with 1.0×10^10^ PFU adenovirus-GSK-3β-RNAi during surgery. UUO and sham procedures were performed as previously described^11^. The study protocol was approved by the Animal Experimentation Ethics Committee of Beijing Friendship Hospital and the NIH Guidelines for the Care and Use of Laboratory Animals were followed.

### Cell Experiments

#### Cell Culture and Treatments

Human proximal renal tubular epithelium (HK-2) cells were derived from a US type culture set (Manassas, VA, USA) and were cultured as previously described^11^. After serum starvation for 12 h, HK-2 cells were incubated with Renalase (1000 ng/mL; Cloud-Clone Corp, Houston, TX, USA) in the absence or presence of TGF-β1 (2 ng/mL; R&D Systems, Minneapolis, MN, USA), TM (20 μg/ml; Yuanyeshengwu, China) or 4-PBA (10 μm/ml, Sigma,USA) for 48 h except as otherwise noted.

#### Transient Cell Transfection

Lipofectamine®2000 transfection reagent (Invitrogen, Burlington, ON, Canada) was used according to the manufacturer's protocol to transfect HK-2 cells with plasmids containing human GSK-3β or GSK-3β siRNA. Cells at 50% fusion degree were placed in fresh medium containing 5% fetal bovine serum 2 h before transfection. The efficiency of gene overexpression was assessed by western blotting.

### Experimental Techniques

#### Evaluation of Renal Fibrosis

To assess the degree of fibrosis, paraffin-embedded renal tissue sections were stained with Masson's trichrome stain or Sirius red stain using standard protocols^11^. Stained sections were examined using an Eclipse E600 Epifluorescence microscope equipped with a digital camera (Nikon, Melville, NY, USA).

#### Immunofluorescence

Immunofluorescence was performed as previously described^11^. The primary antibodies used were: E-cadherin (Cat No. 20874-1-AP, Proteintech, China) and α-smooth muscle actin (α-SMA) (Cat No. 14395-1-AP, Proteintech). Secondary antibodies were conjugated to Cy3 or FITC. Confocal laser scanning microscopy (TCS SP5; Leica, Mannheim, Germany) was used to visualize cells and sections. Scanning confocal microscopy was used to measure fluorescence intensity.

#### Immunohistochemistry

Immunohistochemistry was performed as previously described. The primary antibodies used were: fibronectin (FN) (Cat No. 66042-1-AP, Proteintech) and collagen I (Col-I) (Cat No. 14695-1-AP, Proteintech).

#### Quantitative reverse transcription (qRT-PCR)

Total RNA was isolated using Trizol reagent (Invitrogen) and methyl chloride according to manufacturer's instructions. qRT-PCR was performed as previously described^11^.Primers were designed using Primer Express software v.2.0 (Applied Biosystems) and are listed in Supplementary Table S1. The amplification conditions were: initial denaturation at 95°C for 10 min and denaturation at 95°C for 10s. Each primer pair was annealed at its optimal temperature for 30 s followed by extension at 72°C for 30 s. After normalization to β-actin mRNA, target mRNA levels were calculated.

#### Western Blotting

Western blotting was performed as previously described^11^ using whole cell lysates and kidney tissue homogenates. The primary antibodies used were: fibronectin (FN) (Cat No. 66042-1-AP, Proteintech), collagen I (Col-I) (Cat No. 14695-1-AP, Proteintech), PERK (ab229912; Abcam, USA), ATF4 (ab216839, Abcam), CHOP (ab11419, Abcam), GSK-3β (ab93926, Abcam), p-GSK-3β-Ser9 (ab131097 Abcam), and p-GSK-3β-Tyr216 (Cat No. 29125-1-AP, Proteintech).

#### Activity detection of GSK-3β

Kinase activity was measured by Off-chip Mobility Shift Assay(MSA) in Carna Biosciences. The enzyme was incubated with fluorecence-labeled substrate and Mg(orMn)/ATP. The phosphorylated and unphosphorylated substrates were separated and detected by MSA device. Specific operations were carried out according to the product manual. Where A equals negative control, B equals positive control and C equals test sample. Calculate the percent inhibition of compound as follows: Inhibition (%) = (1-(C-A)/(B-A)) x 100.

### Statistical Analyses

All data are expressed as the mean ± standard deviation (SD). SPSS version 17.0 software (IBM-SPSS, Armonk, N.Y., USA) was used for statistical analysis. One-way ANOVA was used for comparisons between groups, and then the Student-Newman-Keuls test was performed. p < 0.05 was considered to be statistically significant.

## Results

### Role of GSK-3β/Snail in the Anti-Fibrotic Action of Renalase in UUO Model Mice

**Figure 1:**
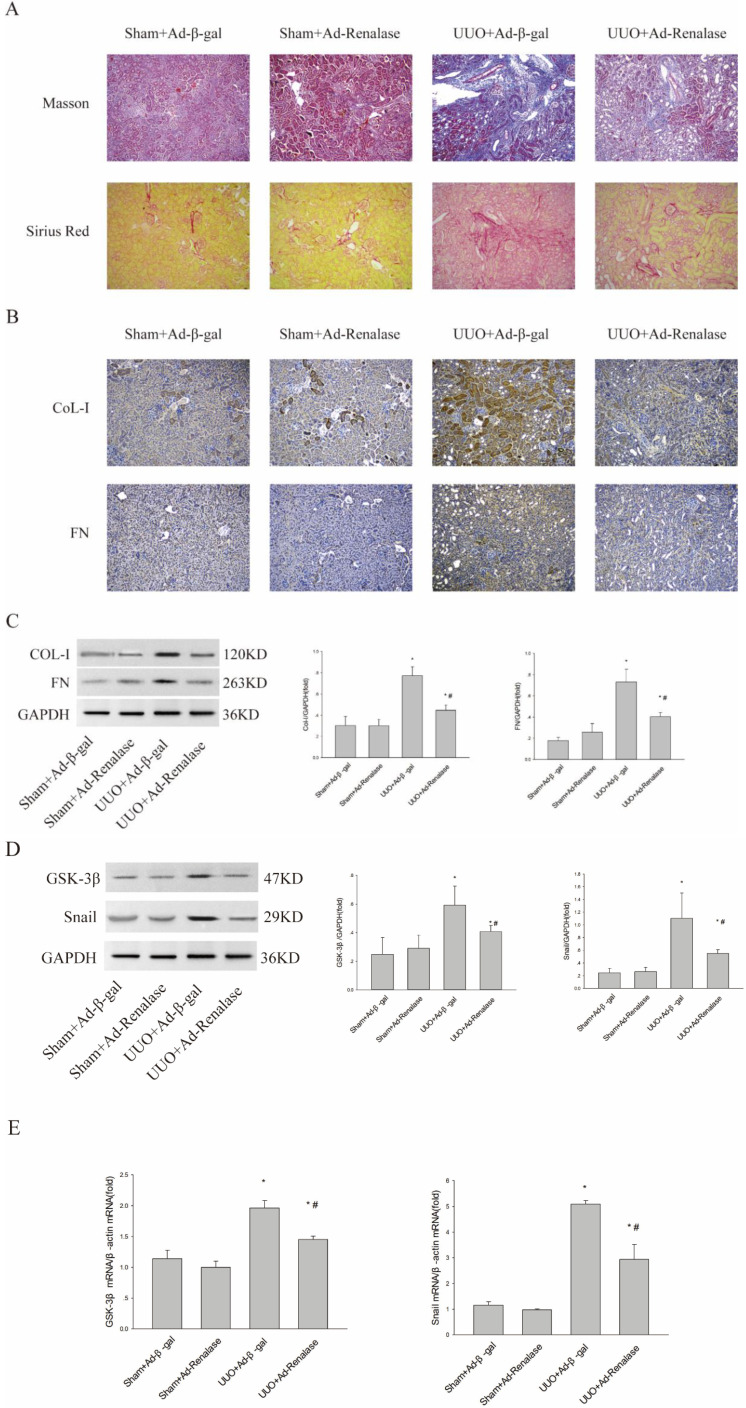
** Role of Renalase and expression of GSK-3β/Snail in UUO mice. (A)** Fibrosis in kidneys of unilateral ureter obstruction (UUO) model mice. Kidney sections from various groups were subjected to Masson's trichrome and Sirius red staining. Representative micrographs showing Renalase-ameliorated renal interstitial fibrosis. Blue represents fibrous tissue in Masson's trichrome staining. Red represents fibrous tissue in Sirius red staining. Magnification 100×. **(B, C)** Western blotting and immunohistochemistry demonstrated increased expression of fibronectin and collagen-I after UUO. The brown/yellow color indicates antibody binding. Magnification 100×. **(D, E)** Western blotting and reverse transcription (RT)-PCR demonstrated increased expression of GSK-3β and Snail in UUO mice and that Renalase reversed the increased expression of GSK-3β and Snail. Results are percentages of control values after normalization to GADPH or β-actin and are the means ± SD of three independent experiments. * p < 0.05, compared with the Sham+Ad-β-gal group; # p < 0.05, compared with the UUO+Ad-β-gal group.

To explore the role of GSK-3β/Snail in the anti-fibrotic action of Renalase in UUO model mice, adenovirus-mediated over-expression of Renalase was used to observe changes in GSK-3β/Snail. First, Masson staining and Sirius red staining showed that Ad-Renalase-treated UUO mice developed significantly less renal fibrosis than Ad-gal-treated UUO mice (ure 1A). Immunohistochemistry and western blotting showed significantly reduced expression of the fibrotic markers, COL-I and FN, in Ad-Renalase-treated UUO mice (Figure 1B, C), indicating that Renalase can reduce renal fibrosis in UUO mice, consistent with previous results^10^. We then assessed the expression of GSK-3β/Snail. There was no change in GSK-3β/Snail expression in Ad-Renalase-treated Sham mice or Ad-β-gal-treated Sham mice (Figure 1D, E). Renalase itself did not, therefore, affect the expression of GSK-3β/Snail. In Ad-β-gal-treated UUO mice, the expression of GSK-3β and Snail was significantly increased at both protein and mRNA levels (Figure 1D, E) compared with Sham mice. We also observed a significant decrease in the elevated levels of GSK-3β and Snail in Ad-Renalase-treated UUO mice (Figure 1D, E). These findings indicated that GSK-3β/Snail was involved in the anti-renal fibrosis action of Renalase.

**Figure 2:**
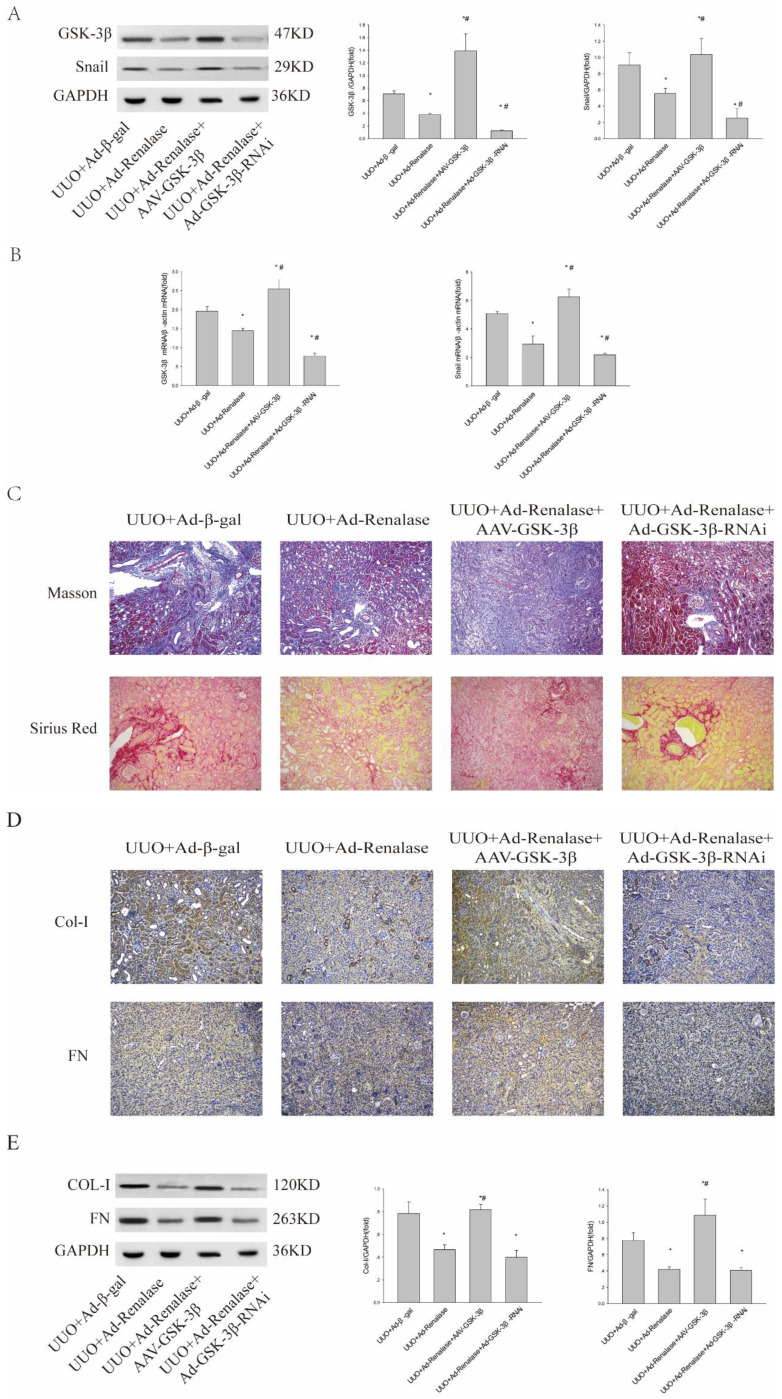
** Role of GSK-3β/Snail in Renalase inhibition of renal fibrosis. (A, B)** Western blotting and RT-PCR revealed that the expression of GSK-3β and Snail increased after over-expression of GSK-3β in the UUO+Ad-Renalase+AAV-GSK-3β group and that the expression of GSK-3β decreased when GSK was inhibited in the UUO+Ad-Renalase+Ad-GSK-3β-RNAi group. **(C)** Kidney sections from various groups were subjected to Masson's trichrome and Sirius red staining. Representative micrographs showing that compared with the UUO+Ad-Renalase group, over-expression of GSK-3β aggravated fibrosis in the UUO+Ad-Renalase+AAV-GSK-3β group while inhibition of GSK-3β reduced fibrosis in the UUO+Ad-Renalase+Ad-GSK-3β-RNAi group. Blue represents fibrous tissue in Masson's trichrome staining. Red represents fibrous tissue in Sirius red staining. Magnification 100×. **(D, E)** Western blotting and immunohistochemistry demonstrated that the expression of fibronectin and collagen-I was increased in the UUO+Ad-Renalase+AAV-GSK-3β group and decreased in the UUO+Ad-Renalase+Ad-GSK-3-RNAi group. The brown/yellow color indicates antibody binding. Magnification 100×. Results are presented as percentages of control values after normalization to GADPH or β-actin and are the means ± SD of three independent experiments. * p < 0.05, compared with the UUO+Ad-β-gal group; # p < 0.05, compared with the UUO+Ad-Renalase group.

**Figure 3:**
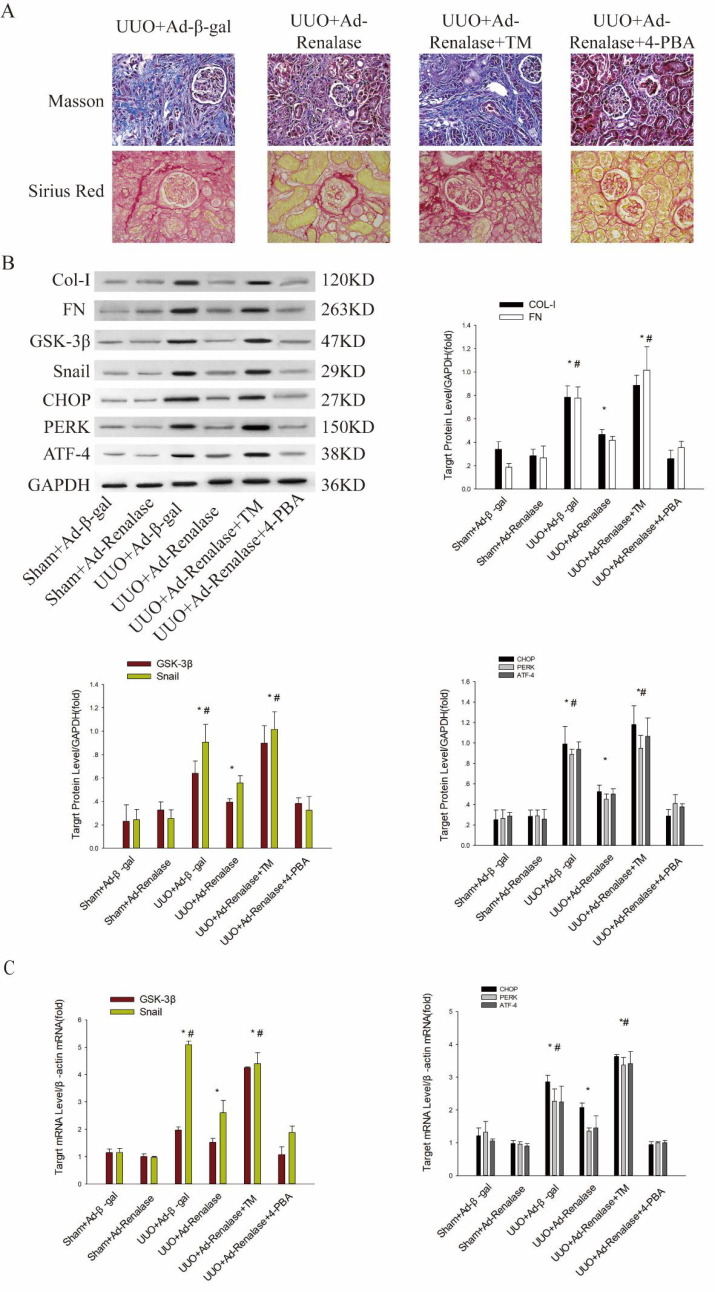

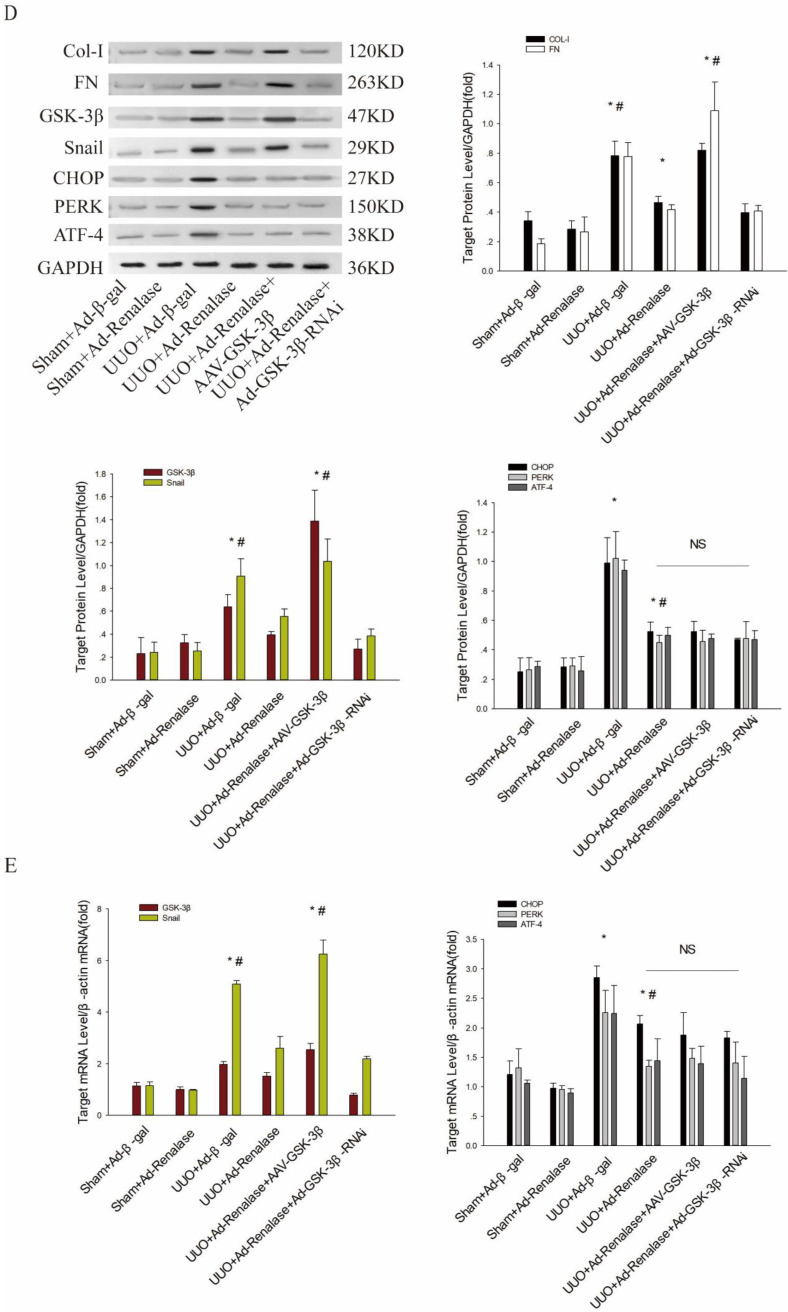
** Role of endoplasmic reticulum stress in Renalase inhibition of renal fibrosis. (A)** Kidney sections from various groups were subjected to Masson's trichrome and Sirius red staining. Representative micrographs showing that compared with the UUO+Ad-Renalase group, activation of endoplasmic reticulum stress in the UUO+Ad-Renalase+TM group aggravated fibrosis and inhibition of endoplasmic reticulum stress did not further reduce fibrosis in the UUO+Ad-Renalase+4-PBA group. Blue represents fibrous tissue in Masson's trichrome staining. Red represents fibrous tissue in Sirius red staining. Magnification 100×. **(B, C)** Western blotting and RT-PCR revealed that compared with the UUO+Ad-Renalase group, when endoplasmic reticulum stress was activated, the expression of Col-I, FN, GSK-3β and Snail was increased in the UUO+Ad-Renalase+TM group. However, when endoplasmic reticulum stress was inhibited in the UUO+Ad-Renalase+4-PBA group, the expression of Col-I, FN, GSK-3β and Snail was not further inhibited. **(D, E)** Western blotting and RT-PCR demonstrated no change in the expression of CHOP, PERK, and ATF4, when GSK-3β was overexpressed in the UUO+Ad-Renalase+AAV-GSK-3β group or knocked down in the UUO+Ad-Renalase+Ad-GSK-3β-RNAi group compared with the UUO+Ad-Renalase group. The results are presented as percentages of control values after normalization to GADPH or β-actin and are the means ± SD of three independent experiments. * p < 0.05, compared with the Sham+Ad-β-gal group; # p < 0.05, compared with the UUO+Ad-Renalase group.

To further clarify the role of GSK-3β/Snail in Renalase inhibition of renal fibrosis in UUO model mice, we overexpressed and knocked down GSK-3β. When GSK-3β was overexpressed by adeno-associated virus (AAV), western blotting and RT-PCR showed GSK-3β/Snail expression was increased (Figure 2A, B). Furthermore, Masson staining and Sirius red staining showed increased fibrosis (Figure 2C) and immunohistochemistry (Figure 2D) and western blotting (Figure 2E) showed significantly increased expression of Col-I and FN in the UUO+Ad-Renalase+AAV-GSK-3β group compared with the UUO+Ad-Renalase group. There was however no significant difference in the levels of fibrotic components between UUO+Ad-Renalase+AAV-GSK-3β and UUO+Ad-β-gal groups. The above results showed that overexpression of GSK-3β reverses the anti-fibrosis effect of Renalase, indicating that the anti-fibrosis effect of Renalase is achieved by down-regulating GSK-3β. Knockdown of GSK-3β in the UUO+Ad-Renalase+Ad-GSK-3β-RNAi group decreased GSK-3Β3β/Snail expression compared with that in the UUO+Ad-Renalase group. However, Masson staining and Sirius red staining showed no significant changes in renal fibrosis (Figure 3C). Western blotting showed that the levels of Col and FN were slightly decreased (Figure 3E), but the difference was not statistically significant, indicating that compared with the UUO+Ad-Renalase group, knockdown of GSK-3β did not further improve renal fibrosis, which further indicated that the anti-fibrosis effect of Renalase was through inhibition of GSK-3β/Snail.

### Mechanism by which Renalase regulates GSK-3β/Snail in UUO Model Mice

Next, we explored the mechanism by which Renalase regulates GSK-3β/Snail expression. We first found that compared with the Sham+Ad-β-gal group, endoplasmic reticulum (ER) stress was significantly activated in the UUO+Ad-β-gal group, with significant increases in protein and mRNA levels of the ER stress markers, CHOP, PERK and ATF4 (Figure 3B, C). Compared with the UUO+Ad-β-gal group, expression of fibrotic markers, COL and FN, was decreased and ER stress activation was significantly reduced at protein and mRNA levels in the UUO+Ad-Renalase group (Figure 3B, C), indicating that ER stress is involved in the occurrence and development of renal fibrosis and that Renalase can inhibit ER stress.

We then explored the effect of ER stress in the anti-renal fibrosis action of Renalase. After intraperitoneal injection of TM, an ER stress activator, renal fibrosis was significantly aggravated in the UUO+Ad-Renalase+TM group compared with the UUO+Ad-Renalase group (Figure 3A). Levels of Col and FN (Figure 3B) and GSK-3β and Snail were significantly increased (Figure 3B, C). When 4-PBA was injected intraperitoneally to inhibit ER stress, the fibrosis was not further improved and the expression of GSK-3β and snail was not further inhibited in the UUO+Ad-Renalase+4-PBA group compared with the UUO+Ad-Renalase group (Figure 3A, B, C), indicating that ER stress is involved in the amelioration of renal fibrosis and regulation of GSK-3β/Snail expression by Renalase.

Finally, we explored the regulatory relationship between ER stress and GSK-3β/snail in the amelioration of renal fibrosis by Renalase. Compared with the UUO+Ad-Renalase group, after overexpression of GSK-3β in the UUO+Ad-Renalase+AAV-GSK-3β group, the anti-renal fibrosis effect of Renalase was almost reversed (as mentioned above; Figure 2C, D, E), while the expression of ER stress markers was still significantly increased (Figure 3D, E). However, when GSK-3β was knocked down in the UUO+Ad-Renalase+Ad-GSK-3β-RNAi group, the expression of ER stress markers did not change significantly (Figure 3D, E), indicating that the change in GSK-3β expression did not affect ER stress status. Therefore, the change in ER stress regulated the expression of GSK-3β/Snail, while the change in GSK-3β did not affect the state of ER stress during the anti-renal fibrosis action of Renalase. This indicated that Renalase regulates the expression of GSK-3β/Snail by inhibiting ER stress to improve renal fibrosis.

### Role of ER stress and GSK-3β/Snail in Renalase alleviation of TGF-β1-induced EMT and fibrosis *in vitro*

**Figure 4:**
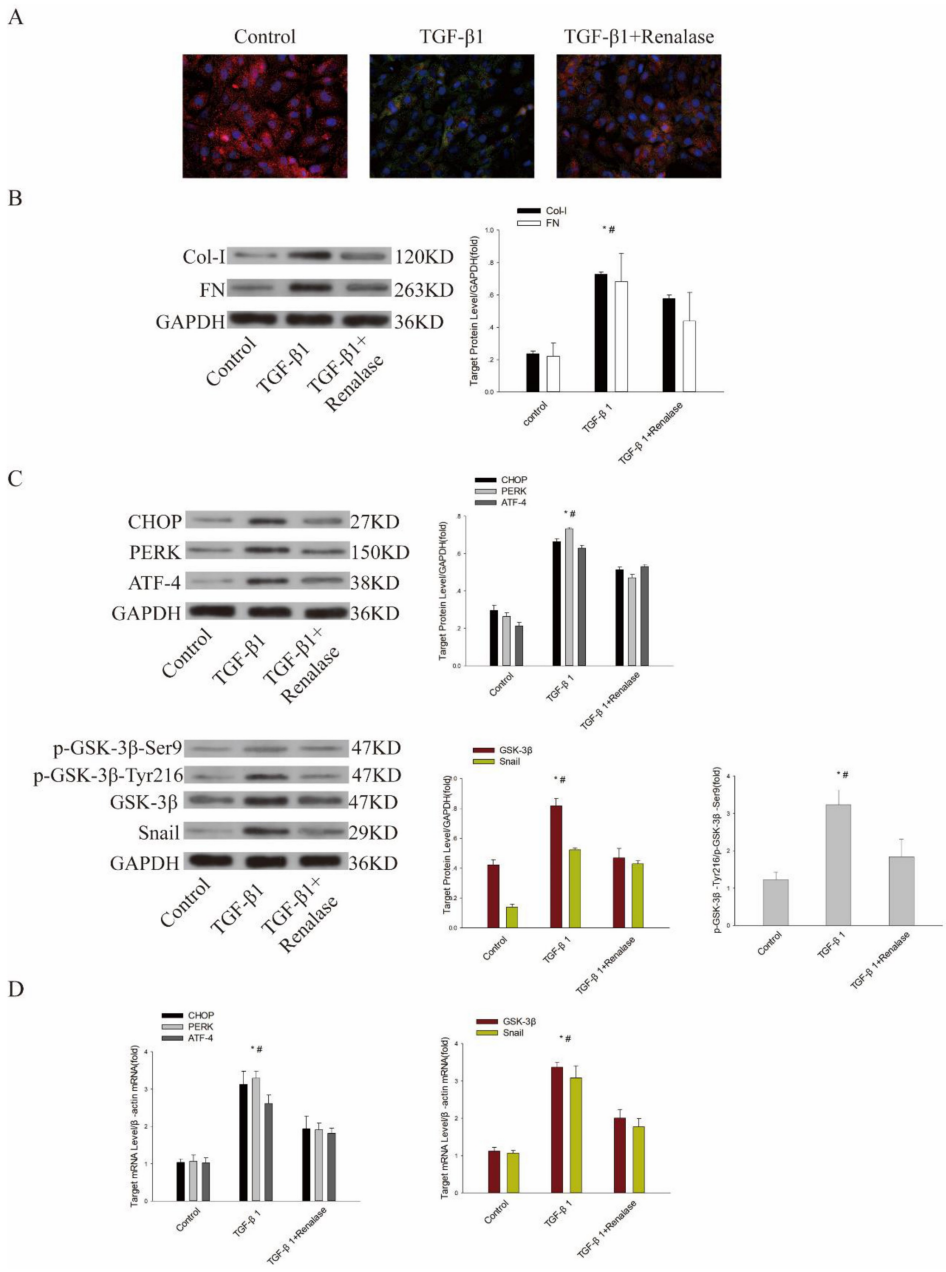
** Role of endoplasmic reticulum stress and GSK-3β/Snail in Renalase amelioration of TGF-β1-induced epithelial to mesenchymal transition (EMT) and fibrosis *in vitro*.** Human proximal tubular epithelial (HK-2) cells were treated with 2 ng/mL TGF-β1 in the presence or absence of Renalase (1000 ng/ml) as indicated for 48 h. **(A)** Immunofluorescence showed that Renalase abolished TGF-β1-induced α-smooth muscle actin (α-SMA) assembly and preserved E-cadherin integrity. Red represents E-cadherin, and green represents α-SMA. Magnification 400×. **(B)** Western blotting demonstrated that Renalase reversed the increased expression of Col-I and FN. **(C, D)** Western blotting and reverse transcription (RT)-PCR revealed that TGF-β1 increased expression of CHOP, PERK, ATF4, p- GSK-3β-Tyr216/p- GSK-3β-Ser9,GSK-3β and Snail, while Renalase reversed these increases. Results are presented as percentages of control values after normalization to GADPH or β-actin and are the means ± SD of three independent experiments. * p < 0.05, compared with control groups; # p < 0.05, compared with TGF-β1+Renalase groups.

Previous studies have confirmed that Renalase can alleviate EMT and fibrosis of human proximal renal tubular epithelial (HK-2) cells induced by TGF-β1^11^. We further explored the role of ER stress and GSK-3β/Snail in Renalase alleviation of TGF-β1-induced EMT and fibrosis *in vitro*. Consistent with previous findings, immunofluorescence showed that the expression of the epithelial cell marker, E-cadherin, was down-regulated and expression of the mesenchymal cell marker, α-SMA, was up-regulated in the TGF-β1 group compared with the control group. Western blotting confirmed that levels of fibrosis markers, COL and FN, were increased. However, in the TGF-β1+Renalase group, Renalase ameliorated these changes (Figure 4A, B). At the same time, the expression of GSK-3β and Snail and the ER stress markers, CHOP, PERK, and ATF4 were significantly increased in the TGF-β1 group compared with the control group. We also observed the increased expression trend of p-GSK-3β-Ser9 in the TGF-β1 group, which could inhibit the activity of GSK-3β, but the difference was not statistically significant. The expression of p-GSK-3β-Tyr216, which could increase the activity of GSK-3β, was significantly increased in the TGF-β1 group. Further, p-GSK-3β-Tyr216/ p-GSK-3β-Ser9 was calculated, and the results showed that p-GSK-3β-Tyr216/p-GSK-3β-Ser9 was significantly increased, indirectly reflecting the increased activity of GSK-3β in the TGF-β1 group compared with the control group. In addition, *in vitro*, TGF-β1 significantly increased the kinase activity of GSK-3β compared with DMSO as negative control and staurosporine as positive control (Table 1). Meanwhile, activated ER stress, GSK-3β/Snail, p-GSK-3β-Tyr216/p-GSK-3β-Ser9 (Figure 4C, D) and elevated kinase activity of GSK-3β (Table 1) were significantly down-regulated after co-culture with Renalase, indicating that inhibition of ER stress and GSK-3β/snail signaling were involved in the antagonism of TGF-β1-induced EMT and fibrosis by Renalase in HK-2 cells.

**Figure 5:**
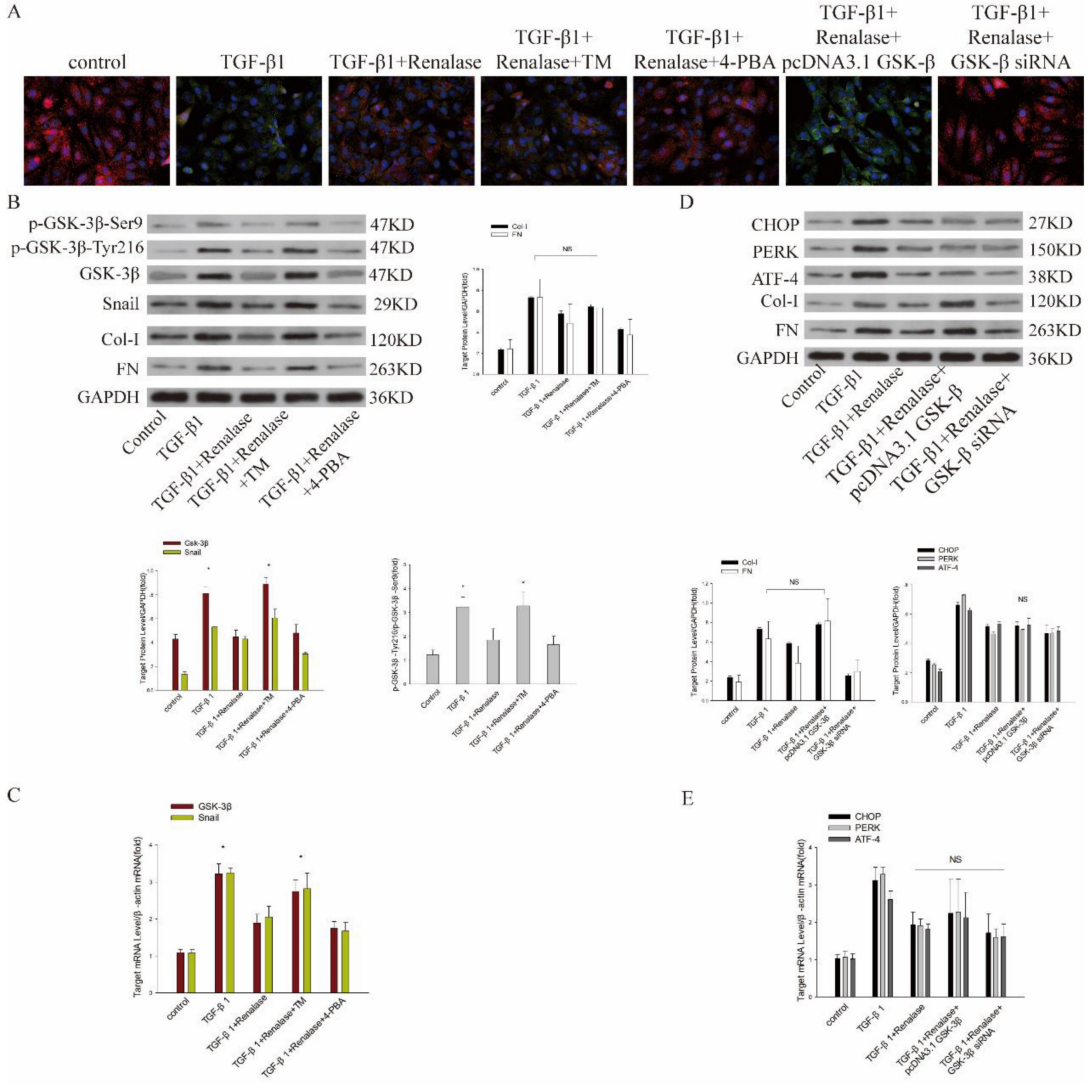
** Mechanism of Renalase antagonism of TGF-β1-induced fibrosis through endoplasmic reticulum stress and GSK-3β/Snail.** HK-2 cells with or without transfection of a plasmid containing human GSK-3β or GSK-3β siRNA were incubated with Renalase (1000 ng/mL) in the absence or presence of TGF-β1 (2 ng/mL), TM (20 μg/ml) or 4-PBA (10 μm/ml) for 48 h. **(A)** Immunofluorescence showed that after activating endoplasmic reticulum stress in the TGF-β1+Renalase+TM group or overexpressing GSK-3β in the TGF-β1+Renalase+pcDNA3.1GSK-3β group, E-cadherin expression was decreased and α-SMA expression increased compared with TGF-β1+Renalase group; that is, the amelioration of TGF-induced fibrosis by Renalase was counteracted. Red represents E-cadherin, and green represents α-SMA. Magnification 400×. **(B)** Western blotting demonstrated that activation of endoplasmic reticulum stress in the TGF-β1+Renalase+TM group did not significantly affect the expression of Col-I and FN relative to the TGF-β1 group. Compared with the TGF-β1+Renalase group, p-GSK-3β-Tyr216/p- GSK-3β-Ser9, GSK-3β and Snail expression were increased when endoplasmic reticulum stress was activated in the TGF-β1+Renalase+TM group. NS: not statistically significant between TGF-β1 and TGF-β1+Renalase+TM groups. * p < 0.05, compared with the TGF-β1+Renalase group. **(C)** Reverse transcription (RT)-PCR revealed that compared with the TGF-β1+Renalase group, the mRNA levels of GSK-3β and Snail were increased when endoplasmic reticulum stress was activated in the TGF-β1+Renalase+TM group. * p < 0.05, compared with the TGF-β1+Renalase group. **(D)** Western blotting demonstrated that over-expression of GSK-3β in the TGF-β1+Renalase+pcDNA3.1GSK-3β group, did not significantly change the expression of Col-I and FN compared with the TGF-β1 group. Compared with the TGF-β1+Renalase group, irrespective of whether GSK-3β was activated in the TGF-β1+Renalase+pcDNA3.1GSK-3β group or inhibited in the TGF-β1+Renalase+GSK-3β-RNAi group, there was no significant change in endoplasmic reticulum stress markers. NS: not statistically significant between groups. **(E)** RT-PCR revealed that compared with the TGF-β1+Renalase group, irrespective of whether GSK-3β was activated or inhibited, there was no significant change in the mRNA levels of endoplasmic reticulum stress markers. NS: not statistically significant between groups.

We then co-cultured cells with Renalase and inhibitors and agonists of ER stress to assess the role of ER stress in the amelioration of fibrosis by Renalase in HK-2 cells. An ER stress agonist increased levels of an ER stress marker protein, while ER stress was inhibited by an ER stress inhibitor (data not shown; Figure S1A, B). This showed that compared with the TGF-β1+Renalase group, when co-cultured with the ER stress agonist, TM, the expression of E-cadherin was decreased and the expression of SMA, FN, and Col-I was increased (Figure 5A, B); that is, the anti-fibrosis effect of Renalase was offset in the TGF-β1+Renalase+TM group, indicating that Renalase can inhibit TGF-β1-induced renal tubular epithelial cell fibrosis by inhibiting ER stress. At the same time, we found that, compared with the TGF-β1+Renalase group, the expression of GSK-3β/Snail, p-GSK-3β-Tyr216/p-GSK-3β-Ser9 and the kinase activity of GSK-3β was significantly increased when ER stress was activated in the TGF-β1+Renalase+TM group, but decreased when ER stress was inhibited in the TGF-β1+Renalase+4-PBA group. However, the degree of decline was not statistically significant (Figure 5B, C; Table 1). These results indicated that ER stress can regulate the expression of GSK-3β/Snail in the anti-fibrosis action of Renalase in HK-2 cells.

Finally, we further verified the regulatory relationship between ER stress and GSK-3β by expressing and inhibiting GSK-3β. After over-expression or inhibition of GSK-3β, the expression of GSK-3β and Snail (data not shown; Figure S1C, D) and the kinase activity of GSK-3β (Table 1) increased or decreased accordingly. Compared with the TGF-β1+Renalase group, GSK-3β overexpression significantly decreased the expression of E-cadherin, increased the expression of α-SMA, FN and Col-I, and counteracted the anti-fibrosis effect of Renalase in the TGF-β1+Renalase+pcDNA3.1GSK-3β group (Figure 5A, D), indicating that Renalase inhibited TGF-β1-induced renal tubular epithelial cell fibrosis by regulating the GSK-3β/snail signaling pathway. Meanwhile, compared with the TGF-β1+Renalase group, irrespective of whether GSK-3β was activated in the TGF-β1+Renalase+pcDNA3.1GSK-3β group or inhibited in the TGF-β1+Renalase+GSK-3β-RNAi group, there was no significant change in ER stress markers (Figure 5D, E), indicating that GSK-3β changes did not affect the ER stress status. In summary, Renalase regulates GSK-3β/Snail by regulating ER stress to inhibit TGF-β1-induced renal tubular epithelial cell fibrosis *in vitro*.

## Discussion

The progression of renal interstitial fibrosis in CKDs, leads to the gradual deterioration of renal function, eventually leading to end stage renal disease (ESRD)^4^. Delaying renal interstitial fibrosis can effectively prevent CKD patients from progressing to ESRD. We have shown that Renalase can improve renal interstitial fibrosis^10^, ^11^, and we have explored its mechanism further in this study. We demonstrated that after UUO and the occurrence of renal interstitial fibrosis, ER stress was elevated and GSK-3β/Snail were highly expressed. When Renalase was overexpressed through an adenovirus, renal fibrosis was alleviated and ER stress and GSK-3β/Snail expression were also significantly decreased. When ER stress was activated or GSK-3β was overexpressed, the effect of Renalase on UUO-induced renal fibrosis was counteracted. At the same time, when ER stress was activated or inhibited, the expression of GSK-3β/Snail changed correspondingly, but no significant change occurred in ER stress when GSK-3β was overexpressed or inhibited, which is consistent with the results of cell-based experiments. The above results indicate that Renalase inhibited GSK-3β/Snail by inhibiting ER stress, thereby delaying progression of renal interstitial fibrosis.

GSK-3β is a constitutively active kinase that is involved in many processes, including embryo development, cell differentiation, apoptosis, transcription and translation^7^. Currently, the reported actions of GSK-3β in organ fibrosis are inconsistent. In liver fibrosis, activation of GSK-3β may improve insulin and leptin resistance in the liver, thereby improving liver fibrosis^12^; however, the increase of GSK-3β expression may promote the progression of liver fibrosis^13^, ^14^. In pulmonary fibrosis, sinensetin can activate GSK-3β to reduce pulmonary fibrosis^15^, but inhibition of GSK-3β can also improve pulmonary fibrosis^16^, ^17^. In cardiac fibrosis, deletion of GSK-3β in cardiac fibroblasts can lead to myocardial fibrosis^18^, and piperine can improve cardiac fibrosis by activating GSK-3β^19^. However, some studies indicate that metallothionein can reduce cardiac remodeling in diabetic patients by inhibiting GSK-3β^20^. Similarly, recent findings of GSK-3β action in renal fibrosis are controversial. On the one hand, activation of GSK-3β can improve renal fibrosis. For example, fingolimod can repress renal fibrosis by inhibiting myofibroblast production and extracellular matrix synthesis by activating GSK-3β in a UUO model^21^.

Moreover, knockout of AKT^22^ or Serum- and glucocorticoid-regulated kinase 1 (SGK1)^23^ can inhibit UUO-induced renal fibrosis by activating GSK-3β, while lithium^24^, angiotensin II^25^ and highly circulating follicle stimulating hormone (FSH)^26^ can promote renal fibrosis by inhibiting GSK-3β. On the other hand, inhibiting GSK-3β can alleviate renal fibrosis. Following renal ischemia-reperfusion in mice, inhibiting GSK-3β can reduce the number of myofibroblasts and alleviate renal fibrosis 27, 28. *In vitro* experiments show that TGF-β1 can promote the expression of GSK-3β in renal fibroblasts, and inhibiting GSK-3β can alleviate fibrosis induced by TGF-β1^28^. LM49 can reduce extracellular matrix deposition by inhibiting the activity of GSK-3β and alleviate renal fibrosis in diabetic nephropathy^29^. In folic acid-injured mice, the occurrence of renal fibrosis can be reduced and the development of acute kidney injury to CKD can be hindered by inhibiting the expression and activity of GSK-3β^30^, ^31^. In this study, we found that the expression of GSK-3β and Snail, a downstream effector of GSK-3β in fibrosis, increased significantly in *in vivo* and *in vitro* models. When Renalase was administered, the fibrosis was alleviated and the expression of both GSK-3β and Snail was decreased. After overexpression of GSK-3β, the anti-fibrosis effect of Renalase was counteracted, indicating that Renalase inhibited renal fibrosis by inhibiting GSK-3β. This is not completely consistent with previous findings. However, the occurrence of renal fibers is the dynamic result of the interaction between many pro-fibrosis factors and anti-fibrosis factors. What role GSK-3β plays in renal fibrosis remains to be further studied.

Previous studies have confirmed that ER stress can be involved in the occurrence and development of renal fibrosis^32^. In this study, consistent with previous findings^33^-^35^, we found that the ER stress was activated in UUO mice and participated in the progression of renal fibrosis. ER stress can regulate the expression of GSK-3β. Kainic acid can activate GSK-3β by activating ER stress, leading to neuron degeneration^36^, and ER stress is involved in the development of Alzheimer's disease by activating GSK-3β^37^. ER stress is also involved in the inflammatory response by activating GSK-3β^38^. Activation of ER stress leads to activation of GSK-3β, which in turn restores the inflammatory response in endotoxin-tolerant macrophages^39^. ER stress participated in myocardial ischemia-reperfusion injury by activating GSK-3β^40^, and inhibiting the appeal process can play a role in myocardial protection. We have also confirmed that Renalase regulates the expression of GSK-3β/Snail by inhibiting ER stress *in vivo* and *in vitro*, thereby delaying fibrosis.

However, this study also has some limitations. Firstly, this study found that Renalase can down-regulate GSK-3β by inhibiting ER stress, thereby antagonizing fibrosis, while our previous study found that Renalase can regulate the expression of ERK^11^, and previous studies have confirmed that GSK-3β can regulate ERK to promote fibrosis^7^. Therefore, whether GSK-3β is involved in the regulation of ERK by Renalase remains to be determined. Secondly, the TGF-β/Smad signaling pathway is crucial to fibrosis^4^ and GSK-3β is involved in the regulation of TGF-β/Smad^7^. We found here that Renalase can delay fibrosis by inhibiting GSK-3β; however, we previously showed no obvious change in Smad during the anti-fibrosis action of Renalase; that is, although Renalase inhibited GSK-3β expression, there was no change in Smad. Why this phenomenon occurs, and whether other signaling pathways are involved remains to be studied. Finally, Snail is a downstream transcription factor of GSK-3β. GSK-3β binds to and phosphorylates Snail at two consensus motifs to regulate two this protein in two ways. Phosphorylation at motif 1 leads to degradation of SNAIL while phosphorylation at motif 2 leads to nuclear export of Snail. In this study, only Snail expression was observed to increase and the specific regulation of this mechanism requires further study.

## Conclusions

Collectively, our results confirmed that Renalase can slow the progression of renal fibrosis by inhibiting ER stress and then downregulating GSK-3β/Snail signaling. These data indicate that exogenous Renalase supplementation may be a promising strategy for slowing or halting CKD progression.

## Supplementary Material

Supplementary figure and table.

## Figures and Tables

**Table 1:** GSK-3β kinase activity measured by Off-chip Mobility Shift Assay.

Kinase	Sample	Signal (conversion %)	Inhibition%
GSK-3β(-)	DMSO	2.53±0.37	
GSK-3β(+)	DMSO^1^	59.16±2.87	
GSK-3β(+)	Staurosporine^2^	1.72±0.35	
GSK-3β(+)	TGF-β1	103.98±5.79	161.19±29.37
GSK-3β(+)	TGF-β1+Renalase	74.82±3.11	127.32±1.12
GSK-3β(+)	TGF-β1+Renalase+TM	103.76±7.44	177.53±3.97
GSK-3β(+)	GF-β1+Renalase+4-PBA	62.36±5.31	105.90±11.98
GSK-3β(+)	TGF-β1+Renalase+pcDNA3.1GSK-3β	130.87±8.26	224.95±11.17
GSK-3β(+)	TGF-β1+Renalase+GSK-3β-RNAi	27.01±4.57	43.81±5.95

1: negative control, 2: positive control.
