# Supplementary material for: Renalase Prevents Renal Fibrosis by Inhibiting Endoplasmic Reticulum Stress and Down-Regulating GSK-3β/Snail Signaling
Source: Int J Med Sci. 2023 Mar 21;20(5):669–81. doi: 10.7150/ijms.82192 (PMC10110476; doi:10.7150/ijms.82192)
Supplement: Supplementary file 1 — Supplementary figure and table. [file ijmsv20p0669s1.pdf]

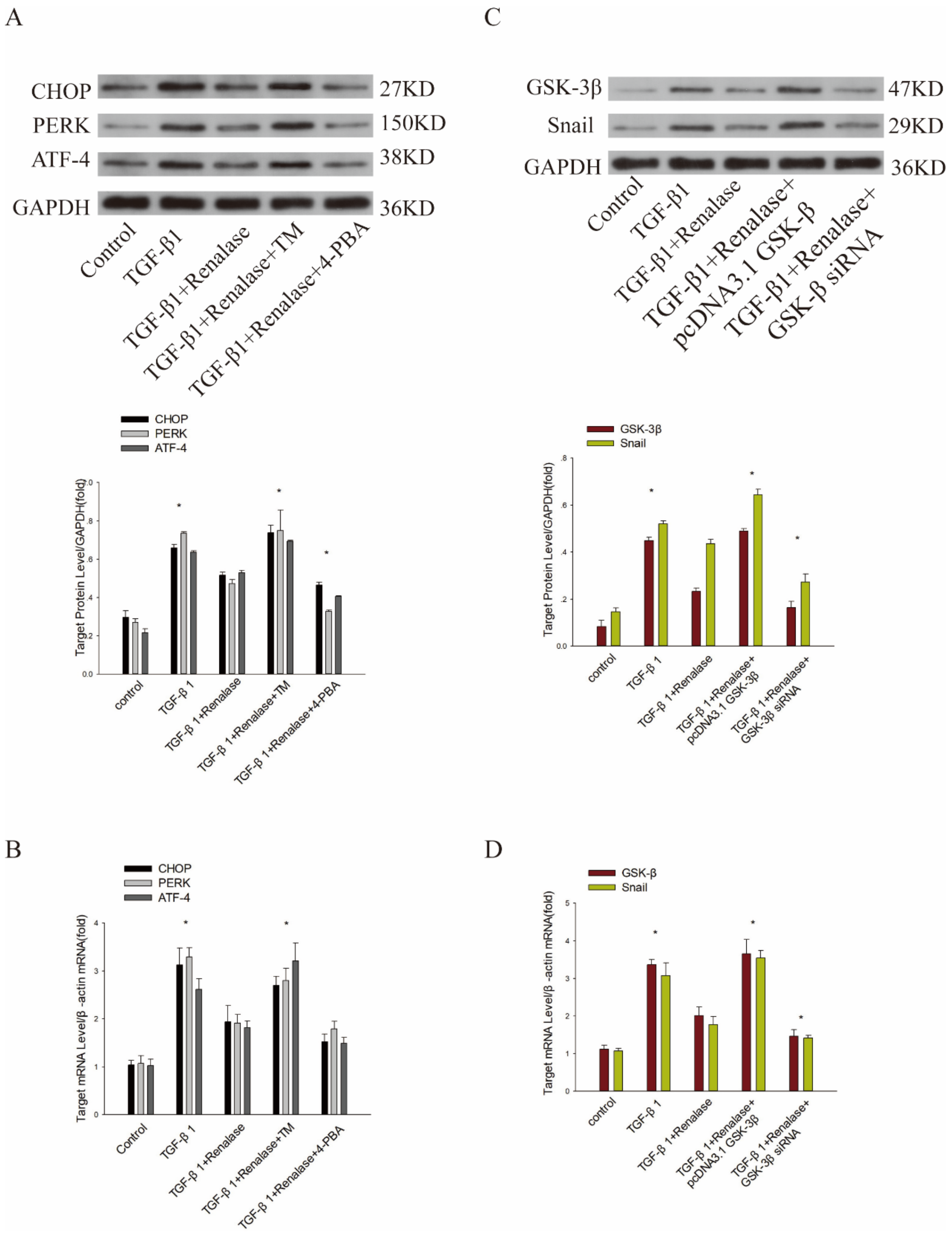


Figure **Supplementary** 1(A,B) Western blotting(WB) and Reverse transcription (RT)-PCR revealed that compared with the TGF-β1+Renalase group, endoplasmic reticulum stress was activated in the TGF-β1+Renalase+TM group and inhibited in TGF-β1+Renalase+4-PBA group . (C,D) Western blotting(WB) and Reverse transcription (RT)-PCR demonstrated that compared with the TGF-β1+Renalase group, GSK-3β and Snail was up-expressed in the TGF-β1+Renalase+pcDNA3.1GSK-3β group and down-expressed in TGF-β1+Renalase+GSK-3β-RNAi group. * $p < 0.05$, compared with the TGF-β1+Renalase group.

Supplementary Table S1

| **Gene** | **Primer** | **Sequence (5'-3')** |
|---|---|---|
| Mouse actin | Forward | CTCCTGAGCGCAAGTACTCT |

| | | |
|---|---|---|
| | Reverse | TACTCCTGCTTGCTGATCCAC |
| Mouse ATF-4 | Forward | CCCTCAGACAGTGAACCCAA |
| | Reverse | GAGTGGAAGACAGAACCCCT |
| Mouse CHOP | Forward | ACTACTCTTGACCCTGCGTCC |
| | Reverse | GGGCCATAGAACTCTGACTGGAA |
| Mouse GSK-3β | Forward | TCCCTTGAGATTCTTCCGA |
| | Reverse | TACTTTGTCAAGCTAACCCC |
| Mouse Snail | Forward | GCTTTTGCTGACCGCTCCAAC |
| | Reverse | CAGCCAGACTCTTGGTGCTTG |
| Homo GAPDH | Forward | TCAAGAAGGTGGTGAAGCAGG |
| | Reverse | TCAAAGGTGGAGGAGTGGGT |
| Homo GSK-3β | Forward | AGACACACCTGCACTCTTCA |
| | Reverse | AGGTGGAGTTGGAAGCTGAT |
| Homo COL-1 | Forward | TGGAGAGGAAGGAAAGCGAG |
| | Reverse | ACCAGCTTCACCAGGAGATC |
| Homo PERK | Forward | GTTGTCGCCAATGGGATAGT |
| | Reverse | CAGCAACCGAAACCTTTATC |
| Homo ATF4 | Forward | GGAAACCATGCCAGATGACC |
| | Reverse | GATCTGGAGTGGAGGACAGG |
| Homo CHOP | Forward | CCCTCACTCTCCAGATTCCAGTC |
| | Reverse | CTAGCTGTGCCACTTTCCTTTCA |
| Homo FN | Forward | TCCCCAACTGGTAACCCTTC |
| | Reverse | CGGGTATGGTCTTGGCCTAT |
| Homo Snail | Forward | TGCTCATCTGGGACTCTGTC |
| | Reverse | GAGGAGAAGGACGAAGGAGC |
